# The Relationship Between Gaze and Classroom Conversation in Primary Education in Chile: A Comparison by Sex and Urban–Rural Context

**DOI:** 10.3390/jemr19040079

**Published:** 2026-07-17

**Authors:** Marco Antonio Villalta-Paucar, Jéssica Verónica Rebolledo-Etchepare, Lautaro Barriga-Carvajal

**Affiliations:** 1 Escuela de Piscología, Universidad de Santiago de Chile, Santiago 9170022, Chile; jessica.rebolledo@usach.cl; 2 Escuela de Psicología, Universidad Bernardo O’Higgins, Santiago 8320000, Chile; lautaro.barriga@ubo.cl

**Keywords:** eye-tracking, primary education, sex, rural area, urban area, conversation, visual attention

## Abstract

This study analyzes the relationship between verbal interaction and eye behavior among 112 primary school students in urban and rural classrooms in Chile, using wireless eye-tracking technology. The results reveal statistically significant differences based on socioeducational context and sex. Linear regression analyses show that gaze is a significantly more robust predictor of class participation in rural contexts (R^2^ adjusted = 0.671) than in urban contexts (R^2^ adjusted = 0.342). Furthermore, eye behavior explained 71% of the variance in male students, compared to 37.7% in female students. While female students focused their attention primarily on teachers, male students relied on a shared visual distribution between the teacher and peers to regulate their participation in class. In conclusion, the gaze acts as a differentiated scaffolding whose importance intensifies in boys and rural environments. These findings suggest distinct maturational trajectories that require teachers to implement visually intentional instructional strategies to ensure communicative efficiency in the classroom.

## 1. Introduction

Looking at the speaker’s face is a predictor of conversational attention and dialogue coordination [1,2,3,4]. Speakers also talk and listen to each other through the gaze [5]. In formal education, verbal interaction between teachers and students is predominant during class [6,7,8,9]. Classes are constituted by verbal and non-verbal exchanges. The advances in eye-tracking have made it possible to delve into the role of gaze in educational dialogue.

Teachers lead the education process in the classroom and consequently, they are the ones who speak the most during class [7], define the topics to be addressed, initiate conversation, or determine the specific forms in which students participate in class [10]. The educational process is interdependent with the organizational and sociocultural contexts of schools. These two aspects are related to the inclusion, leadership and well-being fostered by teachers, which is more often observed in rural schools than in urban schools [11,12,13].

Studies describe several types or patterns of conversation in the classroom, which are related to school culture and inclusion [14,15,16,17], as well as learning [9,18,19,20]. Observations of verbal behavior in students participating in primary education classes indicate that girls are more likely than boys to seek and offer help during peer collaborative work for learning a language [21]. In addition, girls receive more social support from teachers and peers than boys [22], whereas boys tend to learn with more individualistic activities based on element manipulation [23]. However, no differences are found between girls and boys in terms of physical activity and learning in the classroom [24].

Neuroscientific research [25,26] describes different brain development patterns between females and males, which are evident in their early years and equalize in their youth. Nevertheless, gender has been found to be a possible differentiating factor in the motor skills of primary students [27]. For example, a study with kindergarten children (3 to 4 years of age) reports that girls perform better than boys at behavioral and emotional self-regulation [28].

Given the verbal nature of the education process, it is understandable that research on classroom dynamics is predominantly focused on interactions or conversation with teachers and/or peers. However, there are some non-verbal aspects of human communication that are evidently present in classroom interactions, and one of them is the gaze that accompanies verbal participation [29]. The role of the gaze in class has been a topic of interest for ethnographers, psychologists and teachers since the early 20th century [30]. With the development of modern technological tools, it has been possible to study it with more accuracy in different scenarios [31].

Studies in which eye-tracking technology has been applied to analyze the gaze in the classroom have prioritized teachers’ eye behavior [32], for example, to observe differences in the perceptions of experienced and novice teachers [33,34,35,36,37], the gaze distribution of a teacher in a classroom [38], the teacher’s gaze when teaching a subject [39], eye behavior based on teachers’ cultural differences [33,40,41], and dialogues that promote the participation of students in class [42].

Mobile eye-tracking in real classrooms has been employed on 7th grade students—mean age of 12.8 years—from Italian schools to analyze reading patterns that explain learning, finding that gaze patterns that transit between text and image contribute to text retention [43]; and that this comprehensive visual processing can be taught to students as it has a positive effect on reading comprehension [44]. Another study on the eye behavior and dialogue of 9th grade Finnish students during problem solving determined that they focused their gaze mostly on their own and their peer’s notebooks, which leads to inefficient communication between them when solving geometry problems [45]. A recent study about the eye behavior of first year primary students (aged 6 to 9) in real Latin American classrooms established that—regardless of the type of dialogue held in class and the sex of students—rural students look more at the teaching material used by the teacher and their own material than urban students, who gaze more at the teacher [46]. However, research on students’ gaze in real classrooms is scarce.

The study of the gaze in real classrooms, using technological resources, allows for determining the ethnography of communication accurately. This is a field that needs to be explored as most studies have been conducted in laboratory settings and mostly with teachers. Real context research on students is limited, especially in Latin American classrooms. This study addresses the gaze of students in the classroom. The research questions posed are: How do girls and boys distribute their gaze during class activities? Are there differences in students’ gaze distribution and verbal interaction changes depending on the type of classroom (urban or rural)? The objective of the study is to analyze the relationship between verbal interaction and eye behavior of children in classrooms from different Chilean primary education contexts.

## 2. Materials and Methods

This is a descriptive study that employs a mixed methodology and a quantitative approach for the analysis of data collected between 2015 and 2018.

### 2.1. Participants

One hundred and twelve primary students aged 5 to 9 years participated in the study. The students attended 19 Chilean schools located in an urban area—Región Metropolitana—and in a rural area—Región de la Araucanía. The schools selected were characterized by working with students residing in medium and high educational vulnerability areas according to the School Vulnerability Index (IVE) from the Ministry of Education of Chile, i.e., families with socioeconomic needs, without access to most health and recreation services. All the rural schools in this study were located in areas with high educational vulnerability. And all the schools selected reported similar or better achievements than their municipal or regional counterparts during the data collection period (2015–2018). Sample size was defined based on resource limitations, which also influenced data analysis and inference [47]. The cases are described in Table 1.

Regarding teachers, 55 female teachers and 1 male teacher participated in the study. All of them were currently practicing and had experience ranging from 1 to 37 years in primary education classrooms. Rural classrooms are multigrade—characteristic that applies to all Latin American schools [13,48]—i.e., teachers have first and second grade students in a same classroom, with 5 to 12 participants per classroom. Conversely, urban schools only have first grade students in one classroom, with 25 to 40 of them per classroom.

### 2.2. Instruments

Tobii Pro Glasses 2 mobile eye-tracking devices were used for recording the gaze of children (Tobii AB, Danderyd, Sweden). The eye-tracking lens has a frontal camera with 1920 × 1080-pixel resolution, four infrared sensors for detecting and tracking pupils, and an integrated microphone for recording audio, with a sampling range of 100 Hz. The glasses are connected to a recording unit installed in the participant’s pocket. The eye-tracking glasses were put on the students at the beginning of the class and were removed at the end of the class.

A SONY HDR-CX440 recorder in a fixed position was used for the complementary recording of the interaction events in the classroom. The fixed camera was positioned at the back of the classroom facing the whiteboard and behind the students.

### 2.3. Procedure

**Figure 1:**
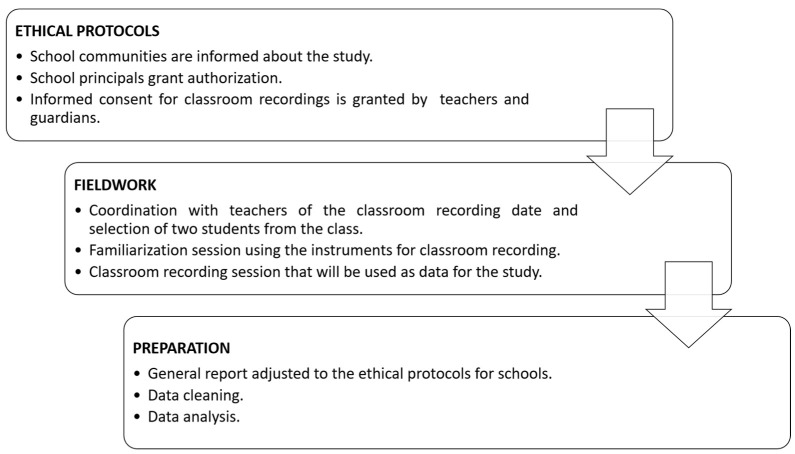
Description of the study’s procedure.

The procedure is defined in Figure 1. The research procedure complied with the ethical protocols approved by a research ethics committee accredited in Chile. Management teams, teachers and guardians were informed of the objectives and procedures of the study. In addition, informed consent and assent letters for conducting recordings during class were signed.

Recordings from real 50-to-60 min classes were collected between 2015 and 2018. To mitigate the equipment logistic limitations and consolidate a robust sample size, a cumulative cross-sectional design was employed along four annual periods (2015–2018). Data equivalence and internal validity were ensured by maintaining strictly homogeneous inclusion criteria for student age and classroom recording settings, specifically educational level and urban/rural schools. The teacher of each group selected two students for using the eye-tracking glasses during class. Selection criteria were the following: the disposition of students to participate in class activities, regular attendance to class, and the informed assent of parents and tutors. Among the participants were several children with special educational needs, whose performance and cognitive development were similar to the group’s average. Since the project only had two devices for data recording, the teachers chose only two children to wear the glasses.

In rural classrooms, students with glasses represented between 17% and 40% of the class, while this percentage varied between 5% and 8% in urban classrooms. Students selected were not different from their classmates in terms of behavior (variable controlled based on the reporting of participating teachers) and school performance (variable controlled based on the student’s grades and assessments). Classes from three subjects were observed—Language, Natural Sciences, and Mathematics—depending on the regular activities in the school calendar. The teachers delivered their lessons with the usual didactic strategies according to their own semester planning. A previous habituation session was held for students to get used to the recording instruments. Some students removed their glasses during class. Such records were not part of the study.

### 2.4. Analysis Procedure

Twenty minutes of the recording were selected. The selection started at the initial part of the class’s development stage, when the teacher introduced the contents of the lesson through diverse didactic strategies. Coding time was set in 20 min, which corresponds to the development stage of the class since, regardless of class duration and type of content imparted, all lessons contain this stage. The development stage is the central part of a lesson, in which the teacher promotes the in-classroom conversation structures that define the participation form between teacher and students. The structures of student–teacher conversation and verbal exchanges about the objectives of the lesson were coded. Exchanges are dialogue units composed of different speakers, and are categorized as Initial, Response and Closure (I-R-C) to achieve a specific communication goal [6]. In this study, only exchanges from the student wearing the glasses were observed and coded, and they were limited to the time segment of his intervention (Table 2).

Exchange coding was conducted using the Videograph version 4.4.2 software –Rimmele, Kiel University, Germany–, program in second units. These exchange time segments were used for coding the student’s eye behavior in this segment. The codification of exchanges and eye behavior was conducted by trained coders, who reached 0.81 and 0.93 of inter-rater reliability coefficient. Eye behavior coding was defined by eye fixations in specific areas of interest. In turn, these areas were established based on previous studies on the teacher’s gaze in the classroom [34,50], and were adapted to the present study in order to classify the gaze distribution of children in the classroom. The areas of interest were classified as follows: teacher, classmates or peers, material used by the teacher, by classmates or own material. In addition, the non-intentional gaze of students when they speak was considered (Table 3). In this way, students’ in-class gaze and speech were recorded.

Eye fixations were coded using the Tobii Glasses Analyzer version 1.171 software. Fixations were set by default at 75 milliseconds and were registered on static AoI maps (Table 3). The software reports fixations on the static AoI in seconds and milliseconds. In a real recording context, students who use the glasses move or interact with the classroom environment, which affects the continuity of eye-tracking; therefore, to ensure data accuracy, records below 60% of gaze capture were excluded to strengthen record reliability.

Data analysis was conducted using descriptive statistics on the variables of interest Type of exchange x gaze fixations, a gaze fixation intersubject contrast test, according to: (1) students’ sex and (2) school area (urban/rural). Given the conditions for defining sample size, sensitivity analysis was selected based on the size of the final sample [47]. The assumption of homoscedasticity was assessed via Levene’s test, and the results were significant, i.e., variance among groups was heterogeneous. Since the assumption of homoscedasticity was not fulfilled, Welch’s *t*-test was applied, which is a robust alternative for heterogeneous variances and unequal sample sizes, thereby ensuring result validity and accuracy. Linear regression was performed taking exchange with teacher and gaze at teacher’s material as dependent variables, and types of exchange x gaze fixation as independent variables.

## 3. Results

Descriptive data (Table 4) show that the highest mean time corresponds to monologue exchanges and gaze at own material (M = 36.13 s), exchange with teacher and gaze at teacher’s material (M = 33.99 s) and exchange with teacher and gaze at teacher (M = 30.50 s). This is possibly related to the goal of the development stage observed in all lessons, which prioritized verbal and visual interaction with the teachers, using the teaching material as support. The lowest mean time corresponds to exchange with the teacher + gaze at peer’s material (M = 1.37 s), exchange with peer and gaze at teacher (M = 2.06 s) and exchange with peer and gaze at teacher’s material (M = 2.49 s); this indicates that dialoguing with peers is not habitual at the beginning of the class development stage. Therefore, the dynamics of the class in this specific moment centered on the teacher and the material presented by her, which possibly introduces the upcoming activities for the class. Nevertheless, emerging dialogues with peers and monologues are also part of this part of the lesson.

The *t*-test for independent samples is applied to compare the 18 variables of exchange and eye fixation among students according to their type of school (rural or urban). In cases in which the Levene test is significant (*p* < 0.05), adjusted t-values are reported (Welch t).

Significant differences between students from urban schools (M = 7.649 s, SD = 20.25) and rural school students (M = 27.491 s, SD = 35.25) are found in exchange with teacher and gaze at own material; in this case, students from urban schools invest significantly less time in this type of exchange and gaze fixation (t (65.68) = −3.44, *p* = 0.001), with a moderate–large effect (d = −0.72).

**Figure 2:**
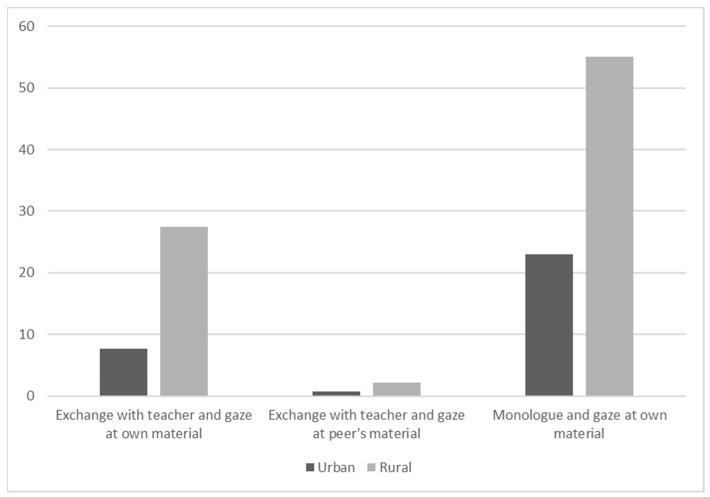
Significant differences in exchange and gaze fixation time among students from urban and rural schools.

Significant differences between urban (M = 0.786 s, SD = 1.85) and rural (M = 2.21, SD = 3.56) students are found in the variable exchange with teacher and gaze at peer’s material, with students from urban school spending significantly less time in this type of exchange and gaze (t (62.14) = −2.48 *p* = 0.008), with a medium effect size (d = 0.53). These results are presented in Figure 2.

Compared to the urban context, rural settings are where students talk more with themselves and with teachers, while fixing their gaze on their own material.

The *t*-test for independent samples is applied to compare the 18 variables of exchange and eye fixation among male and female students. In cases where the Levene test is significant (*p* < 0.05), adjusted values are reported (Welch-t).

Significant differences were observed between boys (M = 12.55 s, SD = 19.30) and girls (M = 5.05 s, SD = 9.51) in the time dedicated to *monologue* and *gaze at the teacher’s material*, with boys investing significantly more time than girls in this type of exchange and gaze fixation (t (80.21) = 2.61 *p* = 0.011), with a moderate effect size (d = 0.49).

Significant differences are found between boys (M = 3.71, SD = 8.38) and girls (M = 1.33 s, SD = 3.03) in the time spent in *monologue* and *gaze at peer’s material*, with boys devoting significantly more time to this type of exchange and eye fixation (t (69.14) = 2.00; *p* = 0.050), and a small–moderate effect size (d = 0.38).

**Figure 3:**
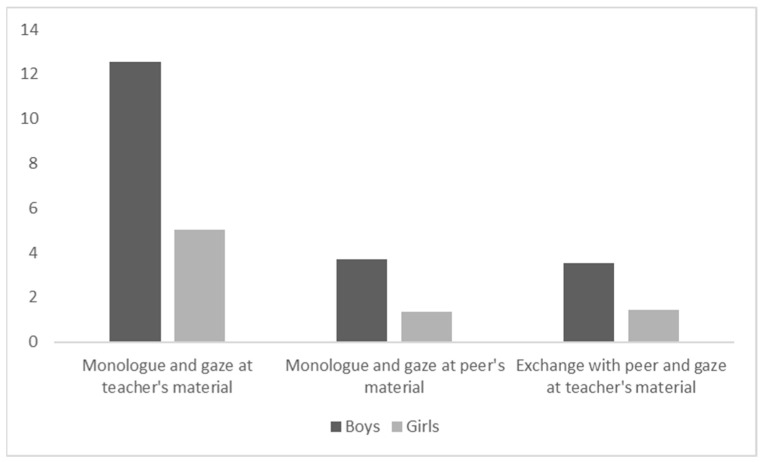
Significant differences in exchange and gaze fixation times between male and female students.

Lastly, significant differences were observed between boys (M = 3.52 s, SD = 7.22) and girls (M = 1.45 s, SD = 2.20) in *exchange with peer* and *gaze at teacher’s material*. In this case, boys spend significantly more time in this type of exchange and eye fixation (t (65.17) = 2.05; *p* = 0.044), with a small-to-moderate effect size (d = 0.39). These results are presented in Figure 3.

According to these results, compared to female students, male students talk more with themselves while fixing their gaze on the teacher’s or peer’s material, and talk with their peers while fixing their gaze on the teacher’s material.

Cause–effect relationships are explored between the exchange and gaze fixation variables by rural/urban area. The independence assumptions of residues, linearity and absence of multicollinearity were first verified, obtaining adequate tolerance values for the Variance Inflation Factor (VIF ≤ 5).

In the multiple regression model, *exchange with teacher and gaze at teacher’s material* was considered as dependent variable, while the predictor variables were (1) exchange with teacher and gaze at own material; (2) exchange with teacher and gaze at peer; (3) exchange with teacher and gaze at peer’s material; (4) monologue and gaze at teacher’s material; (5) monologue and gaze at teacher; (6) exchange with peer and gaze at teacher’s material; (7) exchange with peer and gaze at peer; (8) exchange with peer and gaze at teacher; (9) exchange with peer and gaze at peer’s material; (10) exchange with peer and non-interactional gaze; and (11) exchange with teacher and gaze at teacher.

Regression analyses show differences between urban and rural zones. While in the urban area the model explained 34.2% of adjusted variance (R^2^ adjusted = 0.342), the explanatory capacity of the model rose to 67.1% to predict exchange with teacher and gaze at teacher’s material. In both areas, the strongest and most significant predictor is exchange with teacher and gaze at teacher (urban: β = 0.535, *p* < 0.001; rural: β = 0.658, *p* < 0.001). In urban areas, exchange with peer and gaze at teacher (β = 0.275, *p* = 0.034) also has a direct effect. For rural students, in turn, exchange with teacher and gaze at peer (β = 0.282, *p* = 0.015) emerges as a significant direct influence, suggesting a shared and complex attention dynamic in this environment (see Table 5).

The multiple regression analyses segmented by sex revealed that the model possesses a significantly higher explanatory power for the male group (71% of the adjusted variance (Adjusted R^2^ = 0.710, F = 13.24, *p* < 0.001)) than in the female group (38% of the adjusted variance (adjusted R^2^ = 0.377, F = 4.03 *p* < 0.001)). In both sexes, the variable exchange with teacher and gaze at teacher is the strongest predictor (Male: β = 0.634, *p* < 0.001; Female: β = 0.658, *p* < 0.001). Boys showed more predictive complexity, incorporating exchange with the teacher and gaze at peer (β = 0.236, *p* = 0.011) and exchange with peer and gaze at teacher (β = 0.307, *p* = 0.002) as significant variables. In contrast, for girls, only direct gaze at the teacher during interaction with her was a significant predictor (see Table 6 below).

**Figure 4:**
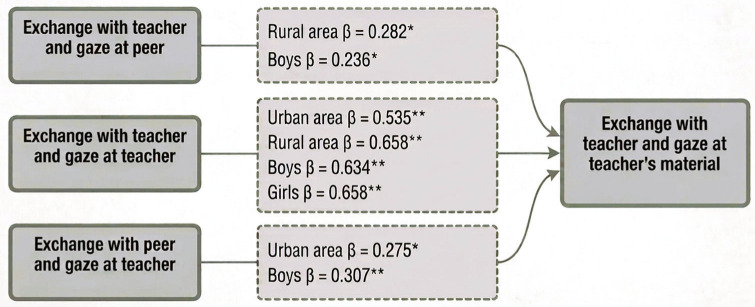
Synthesis of explanatory models of interaction between teacher and their material in the classroom. The values represent standardized beta coefficients (β). Only significant trajectories are shown (* *p* < 0.05; ** *p* < 0.01).

The results confirm that dialogue and visual interaction with the teacher is what best explains maintaining attention to the teacher and the material presented by her in class (see Figure 4).

## 4. Discussion and Conclusions

This study contributes to the understanding of the role of gaze in the participation of students during class. It is observed that in rural classrooms, exchanges with the teacher are significantly more frequent compared with students in urban classrooms. Likewise, gaze fixation is stronger on their own material and that of their peers. This may be explained by the organizational conditions in rural classrooms: fewer students and use of teaching material that allows the teacher to conduct personalized follow-up of didactic activities. In contrast, urban classrooms contain more students, and pedagogical materials are managed in groups in them. In addition, the largest quantity of monologues—dialogues of students with themselves—are observed in rural contexts, and they occur when students are looking at or working with their own teaching material.

Differences in eye behavior have been described between rural and urban education teachers, with rural teachers’ gaze focusing mostly on students and their material [50]. This study has shown that rural students prioritize looking at their own material while holding verbal exchanges with the teacher. In this context, the students’ dialogue with themselves while looking at—and working on—their own material is probably part of the appropriation process of class activities. Nevertheless, this result should be addressed with caution as the differences observed could be attributed not only to geographical location but also to the number of students in the classroom per teacher and the pedagogical organization of the class; therefore, future studies should consider the control of these and other variables. The results suggest that the type of interaction with the teacher and gaze at the teaching material used by every student makes a difference between rural and urban classrooms.

Studies indicate that girls are characterized by offering more help, experiencing more teacher and peer support [21,22]. This study reveals that boys talk more with their peers while gazing at the teaching material used by the teacher. Boys learn through activities of individual nature and hands-on work [23], which explains why boys have more dialogues with themselves—monologues—while looking at the teacher’s and peer’s materials than girls. Data seems to suggest that observing the task and talking are ways in which boys appropriate the activity, although this may possibly affect concentration on the task.

Since the classroom is a predominantly verbal space and time, these data show that boys need, in addition to the verbal information channel, to see the teaching material offered by the teacher and the material used by their classmates more than girls at this age. Girls, in turn, possibly by listening and talking, can participate in class activities in a way that is more independent from the gaze. This has implications for learning styles, with boys possibly requiring more visual elements for sustaining attention in class and consequently participation in the same.

Considering that teachers and materials are inherent to the pedagogical process in the classroom, we seek to answer the following question: what predicts class participation? This is operationalized or expressed in the verbal interaction with teachers and the gaze at the pedagogical material used by them.

The regression analysis by area—rural and urban—highlights the importance of teachers to promote attention and participation in class, as well as the situated and interdependent nature of the rural classroom [51]. Both in urban and rural classrooms looking at the teacher while interacting is key, but this is especially relevant in the rural context, where distracting stimuli are often less and the bond with the teacher tends to be closer [13]. This makes eye behavior a stronger predictor in this setting than in the urban one (67% vs. 34%): in the rural context, gazing at peers while talking to the teacher does matter, while this is not true in the urban context. This suggests that learning in rural schools is more closely connected to others, which is captured through eye-tracking.

Eye behavior strongly explains the participation of boys, but only partly that of girls, who could be using other channels—hearing or internal self-regulation— that eye-tracking cannot fully capture. Boys look at the teacher while talking to their peers, which possibly indicates more dependence on the adult’s visual reference to regulate their social interactions in the classroom. In girls, participation in class seems to depend exclusively on visual and verbal synchrony with the teacher. This supports the idea of more development in self-regulation, which promotes selective attention [28], i.e., they know where to direct focus so class participation is effective.

The results of this study show that verbal and visual participation in the classroom is not a homogeneous phenomenon but rather one mediated by the sociocultural context and the sex of students. Environmental conditions such as fewer distractors, fewer students and closer pedagogical bonds that characterize rural classrooms make eye behavior a strong predictor of the educational interaction promoted by the teacher. In addition, the differences found between men and women strengthen the hypothesis of different maturational trajectories: boys show gaze dependence and speech distributed between peers and the teacher, while girls exhibit a more focused and efficient gaze pattern. These results suggest that for male and rural students, the gaze is an essential scaffolding for effective class participation, validating the need for teaching strategies that prioritize visual contact and attention management for communicative success in class.

Eye behavior is a critical and differentiated element of participation in the classroom. Gaze acts as a much stronger predictor of success in boys and rural contexts than in girls and urban settings. Visual attention is not a universal process but a regulation mechanism that compensates for or potentiates participation depending on maturational trajectory and environmental conditions.

This study presents limitations inherent to research in real context, where, differently from in-lab studies, non-controlled variables may appear. However, these variables can be identified for future studies. For example, the teaching styles of teachers can be differentiating elements of the verbal and visual interaction of students in the classroom. In addition, working with the children who volunteered could introduce bias in responses, as well as the influence of non-interactional gaze on verbal participation in class. Another limitation found is sample size, which is related to the use of recordings from an earlier data collection period. However, the procedural rigor implemented guarantees the high reliability and internal validity of the findings. Furthermore, the difficulty of accessing this type of sample should be considered, which is reflected in the absence of studies with larger samples at the global level. For future research, it is recommended to delve into the conclusions of this study and examine, for example, the cognitive load in the different groups analyzed. It would also be useful to conduct a longitudinal study to verify or inform about the differences in educational trajectories in the classroom’s microprocess.

## Figures and Tables

**Table 1:** Description of cases by sex and primary school location.

	Area		
Sex	Urban	Rural	Total
Male	36	20	56
Female	30	26	56
Total	66	46	112

**Table 2:** Types of verbal exchanges.

Exchange
1. Exchange with the teacher. Conversation structures the teacher uses to develop class topics, which consist of transmission, evaluation, answering questions, promoting the generation of new information, and students’ reflection on the class contents. This integrates exchange categories from other studies [49].
2. Peer exchange. Records of dialogue between students, in which they ask their classmates questions or comment on the class contents.
3. Monologue exchange. Individual work in which students interact with their material using free expression (e.g., speaks about what he is copying from the board).

Source: Created by the authors.

**Table 3:** Areas of interest (AoIs) of students’ eye fixation.

Area of Interest
1. Own material. Fixation on the notebook, pen or material used by the student (boy or girl).
2. Teacher’s material. Fixation on the board, notebook or book that the teacher is using in class.
3. Teacher. Fixation on the teacher’s face, head or hands.
4. Peer (classmate). Fixation on a peer’s face, head or hands.
5. Peer’s (classmate’s) material. Fixation on the notebook, pen or work material of a classmate.
6. Non-interactional. Eye fixation does not cover the previous categories (gaze at ceiling, wall, window, floor, door, etc.).

Source: Adapted from McIntyre, Jarodzka and Klassen [33] and Villalta-Paucar & Rebolledo-Etchepare [46].

**Table 4:** Descriptive data on gaze fixation exchanges in students (*n* = 112).

*n*	Exchange + Gaze Fixation	Minimum	Maximum (s)	Mean (s)	Standard Deviation
1	Exchange with teacher + Non-interactional gaze	0	130.021	13.97	18.84
2	Exchange with teacher + Gaze at peer’s material	0	15.49	1.37	2.77
3	Exchange with teacher + Gaze at teacher	0	237.30	30.50	37.78
4	Exchange with teacher + Gaze at peer	0	77.05	3.41	8.31
5	Exchange with teacher + Gaze at own material	0	156.82	15.80	28.98
6	Exchange with teacher + Gaze at teacher’s material	0	279.10	33.99	53.81
7	Monologue + Gaze at peer’s material	0	37.52	2.52	6.38
8	Monologue + Gaze at teacher’s material	0	91.82	8.80	15.61
9	Monologue + Gaze at own material	0	405.81	36.13	69.84
10	Monologue + Gaze at peer	0	42.17	2.64	6.06
11	Monologue + Gaze at teacher	0	51.51	4.99	8.76
12	Monologue + Non-interactional gaze	0	63.79	8.11	13.42
13	Exchange with peer + Gaze at teacher’s material	0	38.23	2.49	5.41
14	Exchange with peer + Gaze at own material	0	145.92	12.16	23.20
15	Exchange with peer + Gaze at peer	0	135.68	14.21	22.35
16	Exchange with peer + Gaze at teacher	0	26.42	2.06	3.76
17	Exchange with peer + Gaze at peer’s material	0	109.03	8.51	17.40
18	Exchange with peer + Non-interactional gaze	0	146.36	8.56	16.61

Source: Created by the authors.

**Table 5:** Comparison of regression models for visual attention in urban and rural areas.

	Urban Area (*n* = 66)	Rural Area (*n* = 46)
Predictor Variables	β	β
Exchange with teacher and gaze at peer	0.119	0.282 *
Exchange with teacher and gaze at teacher	0.535 **	0.658 **
Exchange with peer and gaze at teacher	0.275 *	0.110
Other predictors	no sig.	no sig.
Adjusted R^2^	0.342	0.671
F	4.08 **	9.36 **

Note. Standardized coefficients are reported (β). * *p* < 0.05, ** *p* < 0.001.

**Table 6:** Comparison of regression models for visual attention by sex.

	Boys (*n* = 56)	Girls (*n* = 56)
Predictor Variables	β	β
Exchange with teacher and gaze at peer	0.236 *	0.122
Exchange with teacher and gaze at teacher	0.634 **	0.658 **
Exchange with peer and gaze at teacher	0.307 **	−0.066
Other predictors	no sig.	no sig.
Adjusted R^2^	0.710	0.377
F	13.24 **	4.03 **

Note. Standardized coefficients are reported (β). * *p* < 0.05, ** *p* < 0.001.

## Data Availability

The data presented in this study are available on request from the corresponding author to protect the confidentiality of the participants.
